# Crystal Structures of the Extracellular Domain from PepT1 and PepT2 Provide Novel Insights into Mammalian Peptide Transport

**DOI:** 10.1016/j.str.2015.07.016

**Published:** 2015-10-06

**Authors:** John H. Beale, Joanne L. Parker, Firdaus Samsudin, Anne L. Barrett, Anish Senan, Louise E. Bird, David Scott, Raymond J. Owens, Mark S.P. Sansom, Stephen J. Tucker, David Meredith, Philip W. Fowler, Simon Newstead

**Affiliations:** 1 Department of Biochemistry, University of Oxford, Oxford OX1 3QU, UK; 2 Clarendon Laboratory, Department of Physics, University of Oxford, Oxford OX1 3PU, UK; 3 Department of Biological Sciences, Oxford Brookes University, Oxford OX3 0BP, UK; 4 Department of Biological and Medical Sciences, Faculty of Health and Life Sciences, Oxford Brookes University, Oxford OX3 0BP, UK; 5 OPPF-UK, Research Complex at Harwell, Harwell Oxford, Didcot, Oxfordshire OX11 0FA, UK; 6 Research Complex at Harwell, Harwell Science and Innovation Campus, Didcot, Oxfordshire OX11 0FA, UK; 7 ISIS Spallation Neutron and Muon Source, Rutherford Appleton Laboratory, Oxfordshire OX11 0FA, UK; 8 School of Biosciences, School of Biosciences, Sutton Bonington Campus, Leicestershire LE12 5RD, UK; 9 OXION Initiative in Ion Channels and Membrane Transport, University of Oxford OX1 3PU, UK

## Abstract

Mammals obtain nitrogen via the uptake of di- and tri-peptides in the gastrointestinal tract through the action of PepT1 and PepT2, which are members of the POT family of proton-coupled oligopeptide transporters. PepT1 and PepT2 also play an important role in drug transport in the human body. Recent crystal structures of bacterial homologs revealed a conserved peptide-binding site and mechanism of transport. However, a key structural difference exists between bacterial and mammalian homologs with only the latter containing a large extracellular domain, the function of which is currently unknown. Here, we present the crystal structure of the extracellular domain from both PepT1 and PepT2 that reveal two immunoglobulin-like folds connected in tandem, providing structural insight into mammalian peptide transport. Functional and biophysical studies demonstrate that these domains interact with the intestinal protease trypsin, suggesting a role in clustering proteolytic activity to the site of peptide transport in eukaryotic cells.

## Highlights

• Crystal structure of the extracellular domains of PepT1 and PepT2
• Modular architecture for a mammalian MFS transporter
• Extracellular domains contain immunoglobulin-like fold and interact with trypsin

## Graphical Abstract

**Figure:**
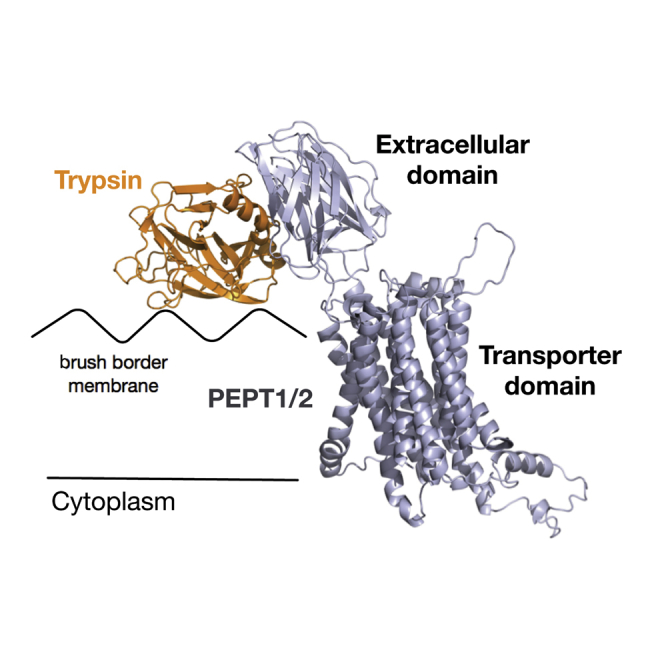

## Teaser

The crystal structure of PepT1 and PepT2 reported by Beale et al. reveals two immunoglobulin-like domains connected in tandem inserted within the canonical major facilitator superfamily fold. Biophysical analyses reveal a specific interaction with trypsin, suggesting a role in clustering proteolytic activity to the site of peptide uptake across the membrane.

## Introduction

In mammals, the plasma membrane transporters PepT1 (SLC15A1) and PepT2 (SLC15A2) mediate the uptake and retention of dietary peptides (Adibi, 1997a; Matthews, 1991). PepT1 and PepT2 are proton-coupled symporters, recognizing di- and tri-peptides on the outside of the cell and utilizing the energy stored in the inwardly directed proton electrochemical gradient (ΔμH^+^) to drive their uptake into the cell (Daniel and Rubio-Aliaga, 2003; Fei et al., 1994). PepT1 and PepT2 also recognize and transport a number of important drug families, including β-lactam antibiotics and anti-cancer agents (reviewed in Brandsch, 2013), and are important targets in the ongoing attempts of the pharmaceutical industry to improve the pharmacokinetic properties of drug molecules (Brandsch, 2013; Smith et al., 2013).

**Figure 1:**
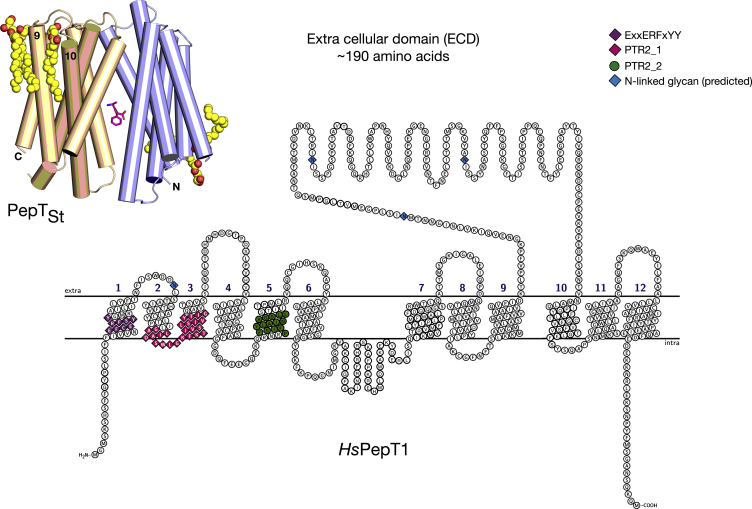
Topology of Mammalian Peptide Transporters Topology diagram of the human plasma membrane peptide transporter PepT1. Conserved PTR2/POT family signature motifs are indicated along with predicted N-linked glycosylation sites, three of which are in the extracellular domain. Inset: Crystal structure of the bacterial homolog PepT_St_ (PDB: 4D2C). The N- (light blue) and C-terminal (wheat) domains are shown as cylinders, with the bound peptide indicating the location of the central peptide-binding site conserved between mammalian and bacterial proteins.

PepT1 and PepT2 are members of the more widely distributed proton-dependent oligopeptide transporter, or POT, family (TC 2.A.17), which are evolutionarily well conserved from bacteria to man (Daniel et al., 2006). Structurally the POT family belongs to the major facilitator superfamily (MFS), with each member containing 12 transmembrane (TM)-spanning α helices arranged into two TM bundles of six that fold to resemble a V-shaped protein that resides within the inner membrane of bacteria and plasma membrane of eukaryotes (Figure 1) (Covitz et al., 1998; Fei et al., 1994; Yan, 2013). The MFS fold can be further subdivided; with each six-helix bundle being constructed from the inversion of two three-helix repeats (Radestock and Forrest, 2011). We recently proposed a structural framework for understanding the transport mechanism within the POT family based on the ability of the triple-helix repeats to work synergistically to alternate the central binding site to either side of the membrane (Fowler et al., 2015). Recent bioinformatics analyses, including sequence-based structure alignments supported by experimental structure validation, further strengthen the importance of the triple-helix repeats. These analyses show that functionally equivalent positions within the different MFS transporters crystallized to date superimpose in three dimensions. This observation has led to the suggestion that evolution within the MFS may have arisen through intragenic duplication and shuffling of these repeats (Madej et al., 2013; Madej and Kaback, 2013).

Crystal structures of bacterial POT family members have revealed a central peptide-binding site that is highly conserved with the mammalian homologs (Doki et al., 2013; Guettou et al., 2014; Solcan et al., 2012). Both in vivo and in vitro assays have demonstrated that while a wide range of peptide substrates are transported by this family, there is conserved substrate specificity, with both bacterial and the mammalian proteins transporting hydrophobic peptides with approximate micromolar affinity and basic peptides with approximate millimolar affinity (Newstead, 2015). Between PepT1 and PepT2 there also exists a difference in overall substrate affinity, with PepT1 having a lower affinity for peptides and PepT2 a higher affinity (Smith et al., 2013). Recent crystal structures of peptide-bound complexes with a bacterial homolog of PepT1 have also suggested that peptides containing extended side chains, such as arginine and lysine, might adopt a less optimal position within the central peptide-binding site that could explain their lower affinity through less favorable interactions (Lyons et al., 2014).

Although the overall sequence identity between the mammalian and bacterial transporters is well conserved within their respective TM domains, epitope tagging analysis, supported by recent structure-based sequence alignments, reveal that in the mammalian PepT1 and PepT2 proteins there exists a significant portion of the protein that is positioned on the outside of the cell (Covitz et al., 1998; Newstead, 2015) (Figure 1). Intriguingly, these domains are completely absent not only in the bacterial members of the POT family but also in the plant and fungal homologs (Parker and Newstead, 2014; Sun et al., 2014) (Figure S1). The functional role of this extracellular domain (ECD) is unknown; however, its presence would suggest the requirement for additional functionality to assist peptide uptake in mammals. Many eukaryotic channels and transporters have evolved to incorporate additional structural domains that extend, constrain, or regulate their function (Barabote et al., 2006). For example, eukaryotic CLC proteins contain intracellular CBS (cystathionine β-synthase) domains that regulate activity in response to nucleotides (Markovic and Dutzler, 2007; Meyer et al., 2007; Zifarelli and Pusch, 2009), whereas the voltage-sensing domains of potassium channels regulate channel opening following membrane depolarization (Pongs and Schwarz, 2010). To date, however, no equivalent domains have been described in detail for any member of the MFS, which forms the largest and most diverse family of secondary active transporters in biology (Reddy et al., 2012).

Here, we reveal that a previously annotated extracellular “loop” in early topology models of the human PepT1 and PepT2 transporters in fact consists of two immunoglobulin-like domains connected in tandem. In vitro binding assays demonstrate that the ECDs interact with the intestinal protease trypsin, potentially answering an interesting observation made in the late 1960s of a specific and tight interaction of trypsin with human intestinal mucosa (Goldberg et al., 1968, 1969a, 1969b). More surprising was the observation that these domains can be removed with no appreciable loss of transport function. These results provide the first structural and biochemical insights into the mammalian SLC15 family, and demonstrate modularity within the MFS that could have important implications for interpreting the function of these proteins in eukaryotic cells.

## Results

### Crystal Structures of the Extracellular Domain of PepT1 and PepT2

Using the crystal structures from the bacterial POT family proteins PepT_So_ (Newstead et al., 2011) and PepT_St_ (Solcan et al., 2012) that share 31% and 21% identity, respectively, with their mammalian homologs, we identified the probable location of the ECD in the PepT1 transporters (Figure S1). We subsequently identified the ECD from *Mus musculus*, consisting of residues 391–580, as being stable and amenable to structural and biophysical analysis (Figure S2). This domain was crystallized and its structure determined using the single anomalous dispersion (SAD) method of phasing using mercury-derivatized protein. The structure was refined to a resolution of 2.1 Å with final *R*_work_ and *R*_free_ of 19.7% and 23.8%, respectively (Table 1). Following extensive screening, crystals from *Rattus norvegicus* PepT2, residues 410–601, were also obtained (Figure S2). The phases were calculated from seleno-L-methionine incorporated protein using the SAD method in space group P3_2_21 (Table 1). A higher-resolution structure in space group P4_1_2_1_2 was obtained using the P3_2_21 crystals as seeds with a single monomer in the asymmetric unit. The final structure refined to a resolution of 2.06 Å with final *R*_work_ and *R*_free_ of 19.9% and 24.0%, respectively.

**Figure 2:**
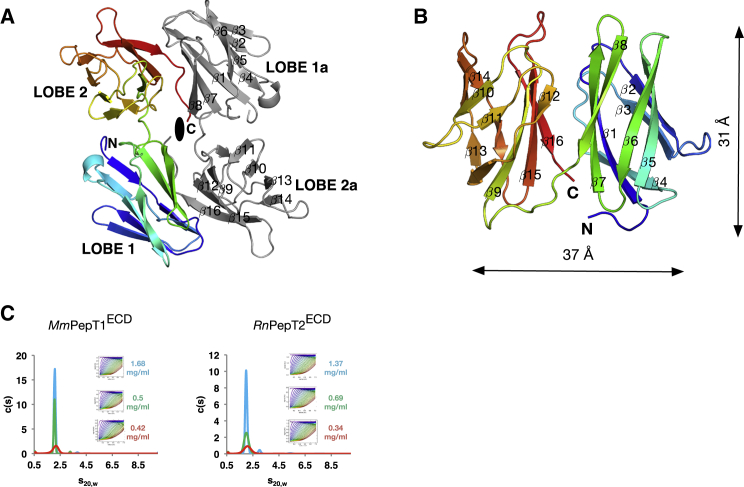
Crystal Structure of the Extracellular Domain from PepT1 and PepT2 (A) The asymmetric unit of *Mm*PepT1^ECD^ containing two monomers related by a two-fold non-crystallographic symmetry axis (black oval). One monomer is rainbow colored from the N terminus to the C terminus; the second is shown in gray with the secondary structure labeled from β1 to β16. (B) Structure of the *Rn*PepT2^ECD^ colored from the N (blue) to the C terminus (red) and with the secondary structure components labeled as for (A). (C) The s^0^_20,W_ values of *Mm*PepT1^ECD^ and *Rn*PepT2^ECD^ from the AUC analysis are 2.16 and 2.22, respectively, consistent with both proteins migrating as a 20-kDa monomer in solution. Inset: the Lamm equation fit profiles for *Mm*PepT1^ECD^ and *Rn*PepT2^ECD^.

The crystallographic asymmetric unit of the PepT1^ECD^ crystal contained two monomers that formed a head-to-tail dimer, related by a two-fold non-crystallographic symmetry axis (Figure 2A), whereas the PepT2^ECD^ construct was crystallized in a monomeric state (Figure 2B). The overall structure of both ECDs consists of two compact β-sandwich immunoglobulin-like folds each comprising two four-stranded β sheets. The β sandwiches are composed of strands in the order 4-1-7-8 and 3-2-5-6, with a short connecting loop between the end of strand β-8 on lobe one and β-9 on lobe two. Despite having only 22% sequence identity the two structures adopt the same overall structure, superimposing with a root-mean-square deviation (rmsd) of 1.83 Å. Analytical ultracentrifugation on the purified ECDs show that both PepT1^ECD^ and PepT2^ECD^ are monomers in solution (Figure 2C). This led us to suspect that the head-to-tail dimer observed in the asymmetric unit was the result of crystallization and that the physiological state of the ECD in PepT1 is monomeric, as shown for the PepT2^ECD^. The bilobal architecture of the ECDs suggested that the two immunoglobulin-like domains have the potential to be highly dynamic, which could represent an important structural and functional difference between PepT1 and pepT2.

### Salt Bridge Interactions Stabilize the Interface between the Immunoglobulin-like Domains in PepT1

**Figure 3:**
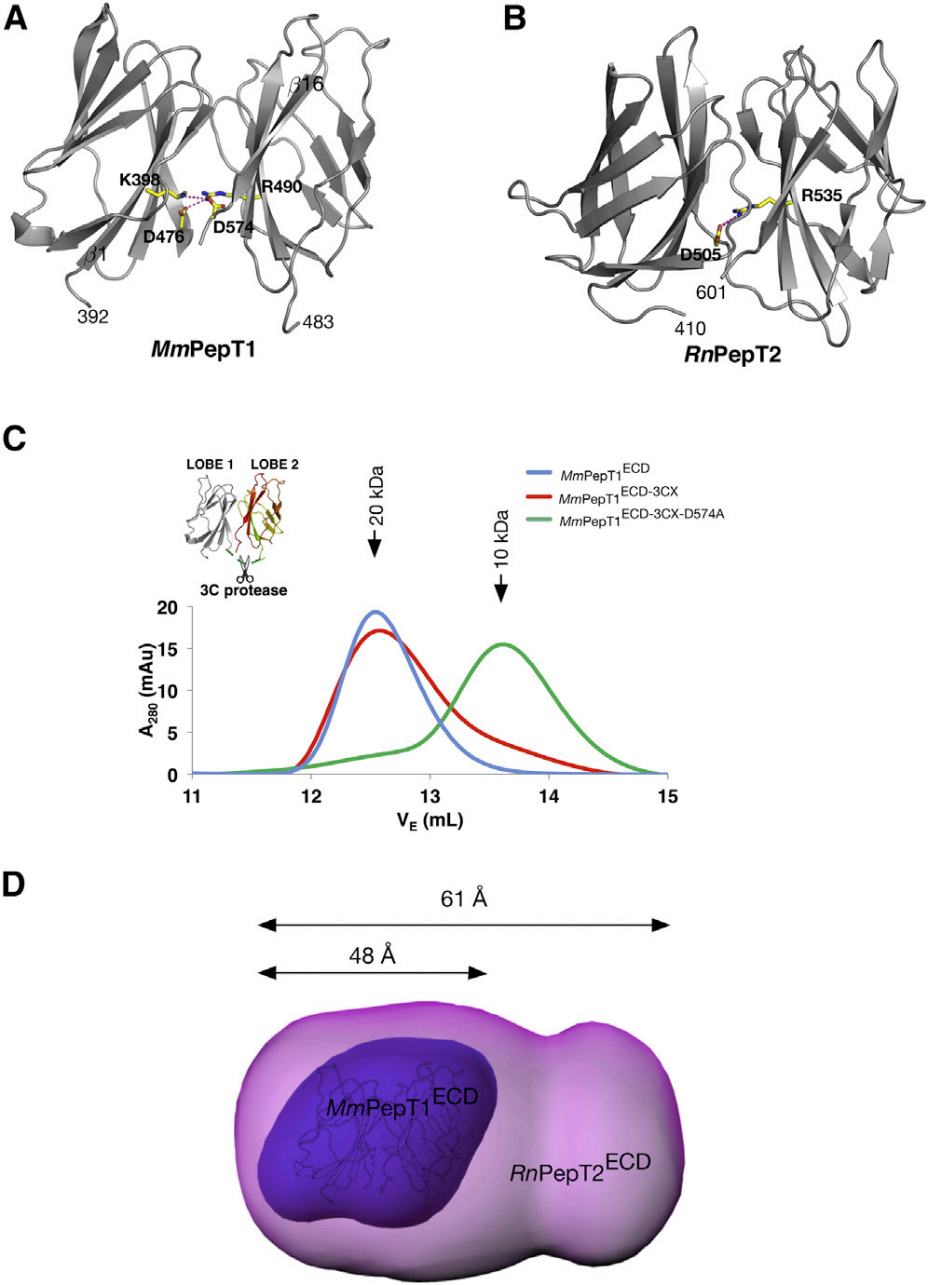
Salt Bridges Stabilize the Interface between the Two Immunoglobulin-like Domains in PepT1^ECD^ and PepT2^ECD^ (A) Structure of PepT1^ECD^ illustrating the two salt bridges, K398 and D574 and R490 and D476, that form an interaction between the lobes. (B) Comparative view in *Rn*PepT2^ECD^, where a single salt bridge is observed between Asp505 and Arg518. (C) Size-exclusion chromatography traces from the *Mm*PepT1^ECD^-3CX experiment. The cleaved *Mm*PepT1^ECD^-3CX constructs elute at the same volume as wild-type, showing that the lobes still interact in solution even after the two lobes are separated. The cleaved *Mm*PepT1^ECD^-3CX-D574A construct, however, elutes in a larger volume consistent with disruption of the interaction. (D) DAMMIN envelopes of *Mm*PepT1^ECD^ (dark purple) and *Rn*PepT2^ECD^ (light purple) calculated from the SAXS data, which show lengths of 48 and 61 Å, respectively, and illustrate the more dynamic behavior of PepT2^ECD^. For scale, a black and white outline of A is overlaid on the *Mm*PepT1^ECD^ envelope.

The crystal structures revealed that in the PepT1^ECD^ there exist two conserved salt bridge interactions stabilizing the interface between lobe 1 and lobe 2, mediated by Asp574-Lys398 and Asp476-Arg490, whereas in PepT2^ECD^ only one equivalent salt bridge interaction is present, between Asp505 and Arg538 (Figures 3A and 3B). To investigate the nature of these interactions, we inserted a 3C protease site into the linker connecting lobe 1 and lobe 2 in PepT1^ECD^ (Figure 3C). Following cleavage by the protease 3C of the purified protein, we observed that the two lobes of PepT1^ECD^ remain associated down a size-exclusion chromatography column. Repeating this experiment with the Asp574Ala variant, however, resulted in the two lobes migrating independently, thus confirming the importance of the salt bridge in holding the two immunoglobulin-like domains together in the compact arrangement shown in Figure 3A.

The same experiment, however, could not be conducted with PepT2^ECD^ as the lobes proved too unstable after cleavage with 3C protease. Therefore, to understand the behavior of PepT2^ECD^ in solution we used small-angle X-ray scattering (SAXS), a method that allows the overall shape of a macromolecule to be modeled at low resolution. Guinier analysis of the scattering data in PRIMUS (Petoukhov et al., 2007) shows that PepT2^ECD^ has a larger radius of gyration (*R*_g_) compared with PepT1^ECD^, 23.1 versus 18.4 Å (Table 2), which suggests an increase in particle size. A shift to larger scattering distances can also be seen in the P(r) distribution and dimensionless *V*_c_ Krakty plot (Rambo and Tainer, 2011) (Figures S3A and S3B), indicating a more elongated structure for PepT2^ECD^. Consistent with this analysis, 3D envelopes of the ECD generated using DAMMIF (Franke and Svergun, 2009) show that PepT1^ECD^ forms a compact shape ∼48 Å in length whereas PepT2^ECD^ is more elongated, approximately 61 Å in length (Figures 3D and S3C). The larger envelope of PepT2^ECD^ indicates that the two immunoglobulin-like domains are structurally more dynamic than the PepT1^ECD^, which is consistent with the loss of the second salt bridge and also the location of Asp505 on the unstructured loop connecting lobes 1 and 2.

### PepT1 and PepT2 Contain a Functionally Independent Extracellular Domain

**Figure 4:**
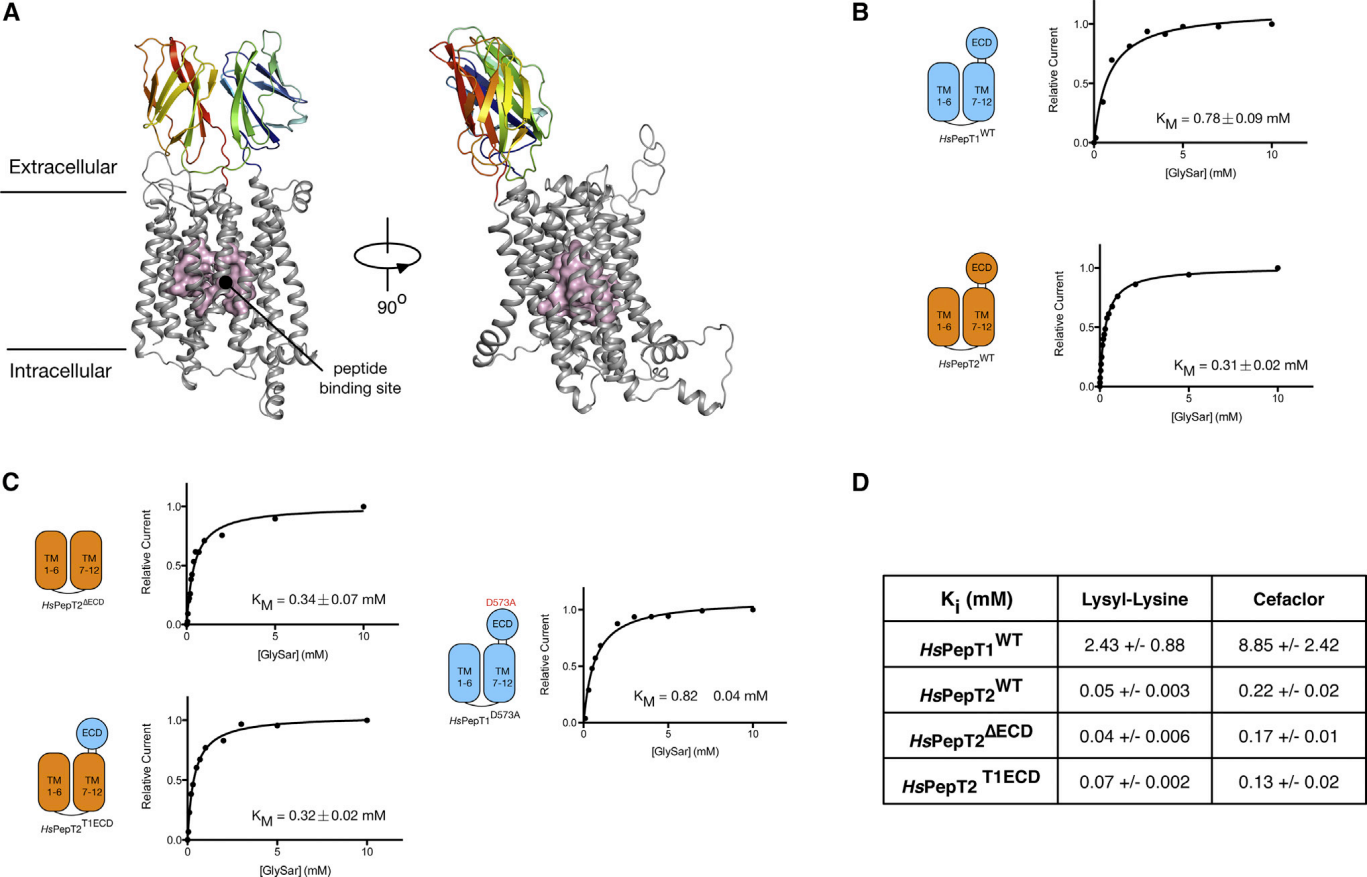
PepT1 and PepT2 are Modular Proteins with Functionally Distinct Domains (A) Homology model of the human PepT1 transporter generated using the crystal structure of *Mm*PepT1^ECD^ (colored blue to red as in Figure 1B) and the recently determined bacterial homolog PepT_So_ representing the transmembrane portion of the transporter (shown in gray). The peptide-binding site is highlighted (magenta). (B) Kinetic analysis of Gly-Sar uptake in human PepT1 and PepT2 using the TEVC method. (C) Kinetic analysis of Gly-Sar uptake in the PepT2^ΔECD^, PepT2^T1ECD^, and PepT1^D573A^ constructs. (D) *K*_i_ values for the different constructs for lysyl-lysine and cefaclor are shown, indicating no effect of removing the ECD on peptide or drug uptake in PepT2.

To date there have been no studies on the role of the ECDs in either of the mammalian peptide transporters. This is largely due to the ambiguity in identifying where this domain was located with respect to the TM helices. The crystal structure of the ECDs from both PepT1 and PepT2 now allow for structure-based homology models of the full-length human transporters to be built (Figure 4A). Molecular dynamics of the models in a palmitoyloleoyl phosphatidylglycerol membrane bilayer suggest that the domains are likely to adopt a vertical orientation sitting to one side of the transporter (Figure S4).

PepT1 and PepT2 are the first MFS transporters to date that have been shown to contain a folded structural domain inserted within the “core” MFS fold (Yan, 2013). This raises the important question of whether the ECDs play any role in transport or determine functional differences between PepT1 and PepT2. To investigate this question, we used the homology models to generate a number of different combinations of chimeric and mutant human PepT1 and PepT2 transporters that had their ECD domains removed (PepT1^ΔECD^, PepT2^ΔECD^) or swapped (PepT1^ECD2^, PepT2^ECD1^). The resulting constructs were expressed in *Xenopus* oocytes and their relative levels of expression examined (Figure S5). We observed that PepT1 was very sensitive to modification at or within the ECD, with only the wild-type (WT) showing stable levels of expression. In contrast, we observed high levels of expression for all of the PepT2 constructs, allowing us to investigate the role of this ECD. Peptide transporters are electrogenic carriers able to concentrate peptides inside the cell using the energy stored in the ΔμH^+^ (Fei et al., 1994). We therefore examined their transport properties using two-electrode voltage-clamp (TEVC) recordings in the *Xenopus laevis* oocytes. This technique measures the inward movement of H^+^ as a function of external peptide concentration. *K*_M_ values for transport of the non-hydrolyzable peptide glycyl-sarcosine (Gly-Sar) were determined for both WT PepT1 and PepT2. The calculated *K*_M_ values confirm the previously noted difference in peptide transport of 0.78 ± 0.09 mM and 0.32 ± 0.02 mM for PepT1 and PepT2, respectively (Figure 4B). However, swapping the ECD region from PepT1 to PepT2 (PepT2^ECD1^) or even deleting it entirely from PepT2 (PepT2^ΔECD^) resulted in no detectable change in the *K*_M_ for uptake of the Gly-Sar peptide, which remained ∼0.3 mM. Additionally, removal of the salt bridge that stabilizes the interface between lobes 1 and 2 of PepT1 (PepT1^D573A^) made no observable difference to the *K*_M_ of Gly-Sar transport (Figure 4C). We also tested whether these constructs interacted differently with the antibiotic cefaclor, a known substrate of PepT1 and PepT2 (Bretschneider et al., 1999; Luckner and Brandsch, 2005). We found that the *K*_i_ of drug uptake in *cis*-inhibition experiments was also unchanged for all the PepT2 constructs, with a *K*_i_ of ∼0.22 mM, compared with ∼0.9 mM for WT PepT1 (Figure 4D). This similarity was also shown with a different non-hydrolyzable peptide, lysyl-lysine. These results indicate that the ECD is a structurally independent unit that has no obvious role in substrate specificity or intrinsic peptide transport function, in PepT2 at least. The immunoglobulin-like structure of the ECDs, however, strongly suggested a possible role in binding an external component that might be present in the intestinal lumen. Therefore, to further investigate the function of the ECDs we undertook a series of binding studies to investigate this possibility.

### The Extracellular Domain Interacts with the Intestinal Protease Trypsin

**Figure 5:**
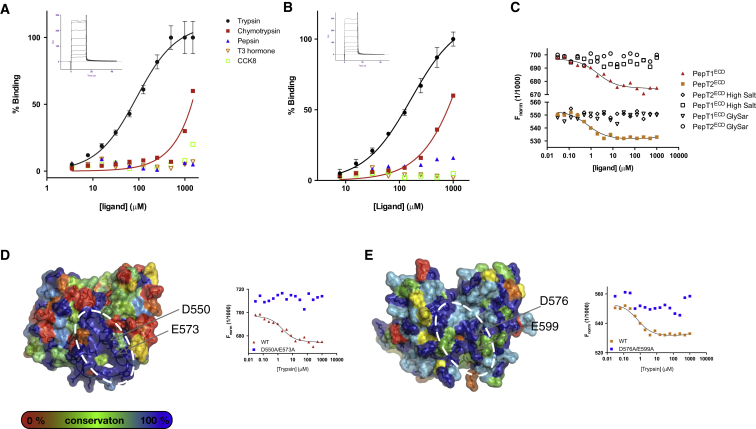
Trypsin Interacts with a Di-acidic Motif on the Extracellular Domain of PepT1 and PepT2 (A) SPR analysis of the *Mm*PepT1^ECD^ interaction with trypsin. Inset: SPR sensorgram used to determine the binding constant. RU, response units. Error bars show the SEM (n = 3). (B) The binding experiment in (A) was repeated with the *Rn*PepT2^ECD^ protein. (C) MST binding analysis reveals no interaction with the Gly-Sar peptide and abolition of trypsin interaction in the presence of high salt. (D and E) Surface representation of (D) *Mm*PepT1^ECD^ and (E) *Rn*PepT2^ECD^ with the sequence conservation from cow, dog, chicken, human, mouse, and rat species mapped from blue to red. A highly conserved patch (indicated by the white dashed ellipse) was identified. Insets: MST binding analysis reveals an important role for D550 and E573 in *Mm*PepT1^ECD^, and D576 and E599 in *Rn*PepT2^ECD^, in mediating the electrostatic interaction with trypsin.

Given the expression of both PepT1 and, to a lesser extent, PepT2 in the intestinal brush border membrane, we thought it possible that the interaction partners may be intestinal hormones, including cholecystokinin (CCK8) and thyroid hormone, which has been reported to decrease the surface expression of PepT1 in Caco-2 cells (Ashida et al., 2002). Another possibility was an interaction with the intestinal proteases that create the di- and tri-peptides that are subsequently recognized by PepT1 and PepT2 for transport into the cell. We therefore screened a panel of potential interaction candidates using surface plasmon resonance (SPR). From this panel of potential ligands we observed binding only for the intestinal protease trypsin with both mouse PepT1^ECD^ (*K*_D_ 80 ± 0.8 μM) and rat PepT2^ECD^ (*K*_D_ 165 ± 0.5 μM) (Figures 5A and 5B). However, we observed no significant interaction between PepT1^ECD^ and chymotrypsin or pepsin. It is possible that one function of the ECDs could be to interact with and accumulate peptides in the vicinity of the transporter, although this would be unlikely given the TEVC data. However, to test this we used microscale thermophoresis (MST), which can measure the interaction with small ligands with transporters in solution (Parker and Newstead, 2014). We did not observe an interaction with the peptide Gly-Sar, but this technique did confirm our previous SPR data showing a specific interaction with trypsin (Figure 5A). The *K*_D_ calculated using this technique, however, was tighter than that observed using SPR, being 8 ± 0.7 μM for PepT1^ECD^ and 6 ± 0.5 μM for PepT2^ECD^. We interpret this discrepancy with the SPR values as being due to the freedom of interaction of a measurement in solution versus immobilization on a chip surface. To examine the nature of the interaction between trypsin and the ECDs, we repeated the MST binding assay with an increased salt concentration of 0.5 M. This resulted in abolition of the interaction, suggesting that the interaction between trypsin and the ECD is mediated through an electrostatic interface.

### Trypsin Recognition Is Localized to a Conserved Di-aspartate Motif on the Extracellular Domain

To identify possible binding sites on the PepT1^ECD^ and PepT2^ECD^ molecules, we mapped the sequence conservation for six different mammalian homologs (Figure S1) onto the crystal structures. We identified two highly conserved charged residues, Asp550 and Glu573 in PepT1^ECD^ and Asp576 and Glu599 in PepT2^ECD^, located on one face of the ECD structure (Figures 5D and 5E). Both of these residues are found in two conserved sequence motifs at the start of strand β15 and at the end of strand β16 in lobe 2 of the mammalian proteins (Figure S6). Removal of these charges abolished the interaction with trypsin (Figures 5D and 5E, insets), further supporting the hypothesis that the function of the ECD is to recruit trypsin to the site of the peptide transport on the plasma membrane. The other face of the ECD, however, does not contain any conserved charged residues, suggesting that this face in unlikely to be important in an electrostatic interaction. Indeed, mutation of several surface residues in mouse PepT1^ECD^ resulted in no substantial impact on the *K*_D_ of trypsin binding compared with WT protein (Figure S7). The involvement of only two conserved residues in the trypsin interaction may also explain the fast binding kinetics observed in the SPR sensorgrams (Figures 5A and 5B, insets), which suggest that the interaction with trypsin is highly dynamic and likely transient, rather than forming a long-lived complex in the intestinal lumen.

## Discussion

### The Modular Architecture of PepT1 and PepT2

Mammalian peptide transport is a physiologically important route to both assimilate dietary nitrogen in the form of small di- and tri-peptides from ingested protein and retain peptides in the body by selective reabsorption in the kidneys (Adibi, 1997b; Matthews, 1975; Matthews, 1991). Although originally identified in 1994, the 3D structures of PepT1 and PepT2 have remained elusive, and to date no crystal structures are available. However, recent crystal structures of several closely related bacterial homologs provide suitable templates to model the TM domain of the mammalian proteins (Newstead, 2015; Terada and Inui, 2012). Nevertheless, the identity of the ECD, originally identified from hydropathy analysis and epitope insertion studies (Covitz et al., 1998), has remained elusive. Our functional and structural analysis shows that the ECD is a fully independent module that has been incorporated by the mammalian members of the POT family to function in protein-protein interactions on the outside of the cell. Additional structural domains are found in many families of membrane transporters (Barabote et al., 2006). However, the locations of the ECDs within the PepT1 and PepT2 structures are particularly intriguing, in that they are inserted *within* the canonical 12-TM helix MFS fold. This is highly unusual; most additional domains in transporters are appended to either the N- or C-terminal end of the polypeptide chain (Markovic and Dutzler, 2007; Meyer et al., 2007; Warmuth et al., 2009). To our knowledge, this study reports the first structure of an additional domain inserted within the core architecture of a transporter. Interestingly the insertion site within PepT1 and PepT2 is the connection between TM9 and TM10, which represents the junction between the two triple-helix repeats that make up the C-terminal bundle within the MFS fold (Radestock and Forrest, 2011). We recently suggested that these repeats operate in a scissor-like motion that control access to the central peptide-binding site during transport (Fowler et al., 2015). The insertion of the two immunoglobulin-like domains of the ECD, with minimal impact on the ability of PepT1 and PepT2 to function as proton-coupled peptide symporters, would appear to support our hypothesis that the two repeats operate in a coordinated but structurally independent manner.

**Figure 6:**
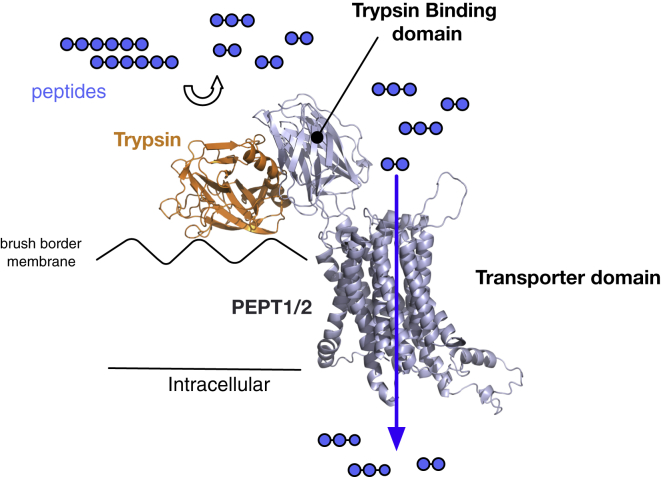
A Model for the Interaction between Trypsin and the Mammalian Peptide Transporters During protein digestion in the small intestine, trypsin transiently docks onto the conserved di-acidic motif on the trypsin-binding domain, localizing the protease to the main site of peptide import on the brush border membrane. Localization would create an increase in the local concentration of arginine- and lysine-containing peptides (shown here as blue circles), which would be expected to increase the efficiency of their uptake into the cell.

However unusual it may be to observe the ECD inserted within the canonical MFS fold, our finding that both the PepT1^ECD^ and PepT2^ECD^ interact with trypsin presents a logical role for these domains in mammalian peptide import. It is interesting to note that as far back as 1968 both trypsin and chymotrypsin were observed to interact with and bind to the mucosa of human small intestine (Goldberg et al., 1968, 1969a, 1969b). The assay conditions used here to investigate the binding between the ECD and trypsin closely resemble the pH and ionic strength found in the small intestine (∼150 mM NaCl and pH ∼6.5) (Fallingborg, 1999; Fordtran et al., 1968), suggesting that the *K*_D_ values reported are likely to be in the physiological range. Furthermore, the *K*_D_ values reported in this study (μM range) are also consistent with the estimated concentration of trypsin in the small intestine, which was reported to be ∼7 μM (Goldberg et al., 1969a). Taken together, these observations support the presence of a physiological interaction between the ECD of PepT1 and PepT2 and trypsin in the human body. Our data show that a conserved di-acidic motif on both PepT1^ECD^ and PepT2^ECD^ presents the most likely interaction site with trypsin. However, the fast binding kinetics show that the interaction is highly dynamic and likely to be transient in nature. We have combined all of the information presented to generate a working hypothesis for how trypsin might interact with PepT1 and PepT2 at the plasma membrane (Figure 6). Our data place trypsin on the opposite side of the ECD to the peptide translocation pathway (Newstead, 2015). In this configuration the binding of trypsin would not obstruct subsequent peptide transport, as the protease would be on the opposite side of the transporter from the peptide-binding site.

Why would PepT1 and PepT2 have evolved to localize trypsin rather than other proteases, such as chymotrypsin? PepT1 and PepT2 play an important physiological role in absorbing small peptides arising from digestion of dietary proteins in the small intestine, as well as in reabsorbing filtered peptides generated from luminal peptidases in the kidney (Adibi, 1997a, 1997b). There is clearly an advantage in tethering a protease to the site of peptide uptake on the plasma membrane, in that the peptides will be locally concentrated at the site needed for their recognition and transport. The localization of trypsin, which recognizes and cleaves the peptide chain at arginine and lysine residues, would therefore increase the concentration of peptides containing these side chains on the outside of the cell directly above the peptide-binding site, and would be expected to improve the efficiency of their uptake through the transporter domain. Indeed, clinical studies of peptide transport in the human body have indicated that the transport of arginine-containing peptides is less efficient (Steinhardt and Adibi, 1986), consistent with our findings from the bacterial homolog of PepT_St_ (Solcan et al., 2012), suggesting that the localization of trypsin is an adaption to increase the concentration of peptides containing arginine and lysine, and therefore improve the transport of these peptides into the cell. We hasten to acknowledge, however, that our data do not unambiguously demonstrate a physiological requirement for trypsin in peptide transport and that our hypothesis will require further in vivo study for it to be supported or refuted.

To our knowledge, this study represents the first structural insight into MFS transporters that exist as multi-domain proteins. Interestingly, a similarly sized ECD is also observed in the SLC22 family of MFS transporters, again inserted between TM9 and TM10 (Kalliokoski and Niemi, 2009), suggesting that other eukaryotic MFS transporters have adopted similar mechanisms to extend their functionality in the cell. The present study and homology models of the human PepT1 and PepT2 transporters therefore not only establish a framework for understanding mammalian peptide uptake, but also show how members of the MFS have evolved to incorporate additional structural domains that expand, augment, or constrain their function in eukaryotic cells.

## Experimental Procedures

### Cloning, Expression, and Purification of the Extracellular Domains from PepT1 and PepT2

PepT1^ECD^ from *M. musculus* (residues 391–580, UniProtKB: Q9JIP7) was cloned into an N-terminal maltose-binding protein (MBP) fusion expression vector, pOPINM (Berrow et al., 2007). PepT2^ECD^ from *R. norvegicus* (residues 410–601, UniProtKB: Q63424) was cloned into a different MBP fusion expression vector, pLou3, a derivative of pMAL-c5 vector with a tobacco etch virus protease site, to remove the MBP and an N-terminal histidine tag. Recombinant protein was produced in *Escherichia coli* strain BL21-DE3. Isopropyl β-D-1-thiogalactopyranoside was used to induce expression of the recombinant genes; cells were harvested following overnight induction at 25°C. WT and mutant proteins were purified to homogeneity using standard protocols for Ni-immobilized metal affinity chromatography- and amylose-based purification of the fusion proteins. Seleno-L-methionine-incorporated PepT2^ECD^ was produced using an auto-inducing medium, PASM-5052 (Studier, 2005).

### Crystallization and Structure Determination

PepT1^ECD^ was crystallized in 20% polyethylene glycol (PEG) 6000, 0.1 M 2-(N-morpholino)ethanesulfonic acid (MES) (pH 6.0), 0.2 M ammonium chloride, at 10 mg ml^−1^ and 4°C using sitting drop-vapor diffusion plates. All crystals were cryo-protected in mother liquor with 25% glycerol and cryo-cooled in liquid nitrogen for data collection. Diffraction data were collected on beamlines I24, I03, and I04 at Diamond Light Source, Harwell, UK. Initial data processing was carried out using the Xia2 pipeline (Winter et al., 2013) to XDS (Kabsch, 2010). Initial phases for PepT1^ECD^ were calculated using SAD with a single mercury-derivatized crystal. The space group was determined to be P2_1_2_1_2_1_. Three mercury sites were initially located using SHELXC/D (Sheldrick, 2010), with their positions further refined and initial phases calculated using SHARP with solvent flattening in SOLOMON (Abrahams and Leslie, 1996). The complete primary structure was assigned in Coot and refined using BUSTER (Blanc et al., 2004) to a final resolution of 2.10 Å and *R*_work_/*R*_free_ of 19.7/23.8 (Table 1).

*Rn*PepT2^ECD^ was crystallized in 0.2 M (NH_4_)_3_ citrate (pH 5.8) and 21% PEG 3350, 10 mg ml^−1^ at 20°C. Initial phases were calculated using Se-SAD data using autoSOL from the PHENIX crystallography suite (Adams et al., 2010). A starting model was built using PHENIX Auto-Build showing three molecules in the asymmetric unit in space group P3_2_21. The complete structure of the three molecules was built in Coot based on this initial map. Refinement of the structure was carried out in BUSTER to a final resolution of 2.81 Å with an *R*_work_/*R*_free_ of 19.7/24.5. To improve the resolution of the PepT2^ECD^ structure, we re-screened the original sparse matrix crystal screens using a seed stock generated from the P3_2_21 crystals. Crystals grew in 0.2 M CsCl_2_ and 15% PEG 3350. A dataset was collected on beamline IO3 at Diamond Light Source and processed using XDS to a resolution of 2.06 Å in a new space group, P4_1_2_1_2. The phases for this new structure were calculated by molecular replacement of the monomeric PepT2^ECD^ structure in Phaser (McCoy et al., 2007). The model was refined in BUSTER to a final *R*_work_/*R*_free_ of 19.9/24.0.

### Homology Models of Human PepT1 and PepT2

Homology models of the human PepT1 (UniProt: P46059) and PepT2 (UniProt: Q16348) transporters were built consisting of the crystal structure of a bacterial peptide transporter, PepT_So_ (Newstead et al., 2011) (PDB: 2XUT) as the template for the transmembrane region, and the crystal structures of the ECD regions inserted within the extracellular loop connecting TMH9 and TMH10. The two extra helices, HA and HB (residues 226–285), which are only present in a subset of the prokaryotic POT family transporters, were removed prior to the sequence alignment. Initial alignment was generated using Probcons (Do et al., 2005), and this was manually refined in Jalview (Waterhouse et al., 2009) to correctly align the functionally important residues identified previously through functional studies on the eukaryotic and mammalian homologs.

### Expression and Functional Characterization of Human PepT1 and PepT2 in *Xenopus* Oocytes

Open reading frames for the human PepT1 and PepT2 proteins were cloned into the pBF *Xenopus* oocyte expression vector, which adds the 5′ and 3′ UTRs of the *Xenopus* β-globin gene. ECD deletions and chimeras were generated using extension overlap PCR. A C-terminal FLAG tag epitope was engineered at the C terminus of these genes to aid detection and quantitation of expression by western blot. mRNA for injection was prepared by in vitro transcription using the AmpliCap SP6 High Yield Message Maker kit (Cellscript). *Xenopus* oocytes were injected with 50 ng of mRNA and incubated at 17°C for 3–4 days before recording. For TEVC recordings, microelectrodes were filled with 3 M KCl and had tip resistances of ∼5 MΩ. Oocytes were voltage-clamped at −50 mV, and a voltage step protocol consisting of 300-ms long pulses from −160 to +60 mV in 10-mV increments was used to test oocyte stability and record currents. ND96 solution was used as the bath solution (95.4 mM NaCl, 2 mM KCl, 1.8 mM CaCl_2_, 5 mM HEPES [pH 7.5] with NaOH). Test solutions consisted Gly-Sar at desired concentrations in ND96 solution. Transport-associated currents were estimated by subtracting currents recorded on ND96 solution only from those in the presence of substrate for each experiment. The currents at each Gly-Sar concentration were normalized to the maximal current (taken as current observed with 10 mM Gly-Sar) for each oocyte and the current at −120 mV at each Gly-Sar concentration used in non-linear regression analysis. Data were analyzed using Clampfit 10.4.0.36 (Axon Instruments) and non-linear regression analysis was performed on data from single oocytes using GraphPad Prism (version 6.04 for Mac, GraphPad Software). Acquired data were fitted to the Michaelis-Menten equation *I* = (*I*_max_[Gly-Sar])/(*K*_M_ + [Gly-Sar]), where current was used in the place of velocity to calculate an apparent *K*_M_ for data from each oocyte. Average *K*_M_ was then taken as the mean of the *K*_M_ values from individual oocytes expressing the specific construct and displayed as mean ± SEM. Background currents were measured in uninjected oocytes 4 days after incubation. Inhibition experiments with the β-lactam antibiotic cefaclor and the dipeptide lysyl-lysine determined *cis-*inhibition of [^3^H]-D-Phe-L-Gln uptake by increasing concentrations of cefaclor or lysyl-lysine, using the method of Pieri et al. (2009). 10 ng of mRNA was injected into each oocyte.

### Small-Angle X-Ray Scattering

SAXS data were collected at the P12 beamline (PETRA III, Hamburg) at a wavelength of 1.24 Å. Scattered images were collected on a PILATUS 2M detector (Dectris) at 297 K. Twenty images were taken while the sample was continually passed through a quartz capillary. Samples and buffers were prepared using the same protocol used for the area under the curve (AUC) analysis. A concentration series was collected for each sample from 5 to 0.15 mg ml^−1^. Raw images were individually examined using PRIMUS (Konarev et al., 2003), and images that showed radiation damage were excluded from the final averaged curves. No concentration-dependent scattering was observed in for PepT1^ECD^. Merged curves were created for PepT2^ECD^, as an increase in scattering was observed as a function of concentration in the low scattering angles. To interpret the scattering data, 20 3D models of each ECD were created using DAMMIF (Franke and Svergun, 2009), which were then aligned, clustered, and averaged using DAMAVER (Volkov and Svergun, 2003). DAMMIN (Svergun, 1999) was then used to compare the averaged model against the raw data to ensure a good fit as evaluated using reduced χ^2^ values. Envelopes of the models were generated using Sculptor (Birmanns et al., 2011).

### Surface Plasmon Resonance

SPR experiments were carried out using a BIAcore T200 instrument (GE Healthcare). Experiments were performed at 20°C in 25 mM MES (pH 6.5), 100 mM NaCl, 10 mM CaCl_2_, 0.005% Tween 20, 2 mg ml^−1^ dextran, and 1 mg ml^−1^ salmon sperm DNA (Sigma-Aldrich). Either PepT1^ECD^ or PepT2^ECD^ were immobilized on a CM5 chip (GE Healthcare) by amine coupling (GE Healthcare kit) to a total of 1,000 response units. A concentration series of ligand (1000, 500, 250, 125, 62.5, 31.2, 15.6, and 7.81 μM) was injected over the ECD-coated chip for 45 s at 90 μl min^−1^, followed by a 30-s dissociation time. The chip surface was then regenerated with 2 M NaCl for 30 s. Specific binding of trypsin was obtained by subtracting the response from a blank surface from that of the ECD-coated surface. The kinetic sensorgrams were fitted to a global 1:1 interaction model, allowing determination of the dissociation constant, *K*_D_, using BIAevaluation software 1.0 (GE Healthcare).

### Microscale Thermophoresis

MST was carried out using a Monolith NT.115 instrument (NanoTemper). Experiments were performed at 22°C in 25 mM MES (pH 6.5) and 100 mM NaCl. Both PepT1^ECD^ and PepT2^ECD^ were mutated to change a surface-exposed serine to a cysteine (PepT1^ECD^-S437C and PepT2^ECD^-S427C) and labeled with the blue maleimide labeling kit MO-L006 (NanoTemper). A range of concentrations of the required ligand (range 0.03–1,000 μM) was incubated with 1.5  μM of purified labeled protein. The sample was loaded into the NanoTemper glass capillaries and microthermophoresis was carried out using 100% LED power and 80% MST. *K*_D_s were calculated using the mass action equation via the NanoTemper software from duplicate reads of triplicate experiments.

## Author Contributions

S.N. conceived the study. J.H.B., J.L.P., L.E.B., R.J.O., and S.N. cloned and purified the protein. J.H.B. and S.N. crystallized the protein and collected the X-ray diffraction data, solved the structures, and built and refined the models. A.L.B. and S.J.T. designed, performed, and analyzed the TEVC experiments. A.S. and D.M. designed, performed, and analyzed the radioactive uptake assays. J.H.B. and D.S. designed, performed, and analyzed the AUC experiments. J.H.B. and J.L.P. designed, performed, and analyzed the biochemical and binding assays. F.S., M.S.P.S., and P.W.F. built the full-length PepT1 and PepT2 homology models and designed, performed, and analyzed the molecular dynamics experiments. J.L.P. and S.N. wrote the paper.

## Figures and Tables

**Table 1:** Data Collection and Refinement Statistics for *Mm*PepT1^ECD^ and *Rn*PepT2^ECD^

	*Mm*PepT1^ECD^	*Mm*PepT1^ECD^-Hg^a^	*Rn*PepT2^ECD^-Se^a^	*Rn*PepT2^ECD^
Space group	P2_1_2_1_2_1_	P2_1_2_1_2_1_	P3_2_21	P4_1_2_1_2_1_
Cell dimensions				
*a*, *b*, *c* (Å)	53.48, 70.33, 111.22	53.55, 70.37, 111.16	95.75, 95.75, 165.93	43.1, 43.1, 220.1
α, β, γ (°)	90, 90, 90	90, 90, 90	90, 90, 120	90, 90, 90
Wavelength (λ)	0.968	1.006	0.979	0.976
Resolution (Å)	43–2.10 (2.19–2.10)	70–2.85 (3.05–2.85)	58–2.81 (2.96–2.81)	43–2.06 (2.12–2.06)
*R*_merge_	5.0 (79.8)	17.2 (75.6)	15.4 (109)	6.6 (67.1)
Mn*I*/σ*I*	16.4 (2.7)	15.1 (4.1)	12.7 (2.3)	11.5 (2.3)
CC_1/2_^b^	99.9 (48.0)	99.7 (91.0)	99.9 (69.7)	99.8 (63.9)
Completeness (%)	99.5 (99.0)	99.4 (99.7)	99.9 (99.9)	99.3 (99.5)
Redundancy	4.8 (4.8)	14.0 (14.5)	9.9 (10)	4.1 (4.3)
*R*_cullis_ (%)		69.2	45.1	
Phasing power^c^		1.492	2.468	
Resolution (Å)	43.6–2.10		58.6–2.81	40.1–2.06
No. of reflections	24, 975		22, 048	13, 635
*R*_work_/*R*_free_	19.7/23.8		19.7/24.5	19.9/24.0
Ramachandran favored	96.6		92.5	96.3
Ramachandran outliers	0.53		0.17	0
Rmsd				
Bond lengths (Å)	0.010		0.010	0.010
Bond angles (°)	1.18		1.31	1.25

a For details on derivatization, see Experimental Procedures.

b Mn(*I*) half-set correlation as reported by Aimless.

c Phasing power = rms(|*F*_H_|/((*F*_H_ + *F*_P_) − (*F*_PH_))).

**Table 2:** SAXS Data Statistics for *Mm*PepT1^ECD^ and *Rn*PepT2^ECD^

	*R*_g_ (Å)	*I*(0)/Conc (mg ml^−1^)	*V*_p_ (nm^3^)	*D*_max_ (Å)	*V*_c_ (Å^2^)	Mass (kDa)
*Mm*PepT1^ECD^	18.4 ± 1.7	8.6 ± 0.0	33.4	64.3	197	17.1
*Rn*PepT2^ECD^	23.0 ± 2.8	14.9 ± 0.0	45.4	73.1	238	20.1

The *R*_g_, *I*(0)/Conc, *V*_p_, and *D*_max_ were calculated in PRIMUS. The *V*_c_ and particle mass were calculated in ScÅtter, and show that *Rn*PepT2^ECD^ has a larger radius of gyration (*R*_g_) in solution, indicating a more flexible arrangement for the two lobe domains.

## Accession Numbers

The atomic coordinates have been deposited in the PDB under accession codes PDB: 5A9D (*Mm*PepT1^ECD^) and PDB: 5A9H and 5A9I (*Rn*PepT2^ECD^).

## Supplemental Information

Document S1. Figures S1–S7

Document S2. Article plus Supplemental Information
