# Supplementary material for: Crystal Structures of the Extracellular Domain from PepT1 and PepT2 Provide Novel Insights into Mammalian Peptide Transport
Source: Structure. 2015 Oct 6;23(10):1889–99. doi: 10.1016/j.str.2015.07.016 (PMC4597091; doi:10.1016/j.str.2015.07.016)
Supplement: Document S1. Figures S1–S7 [file mmc1.pdf]

Structure, Volume *23*

Supplemental Information

Crystal Structures of the Extracellular Domain from PepT1 and PepT2 Provide Novel Insights into Mammalian Peptide Transport

John H. Beale, Joanne L. Parker, Firdaus Samsudin, Anne L. Barrett, Anish Senan, Louise E. Bird, David Scott, Raymond J. Owens, Mark S.P. Sansom, Stephen J. Tucker, David Meredith, Philip W. Fowler, and Simon Newstead

Supplementary Figures

Crystal structures of the extracellular domain from PepT1 and PepT2 provide novel insights into mammalian peptide transport.

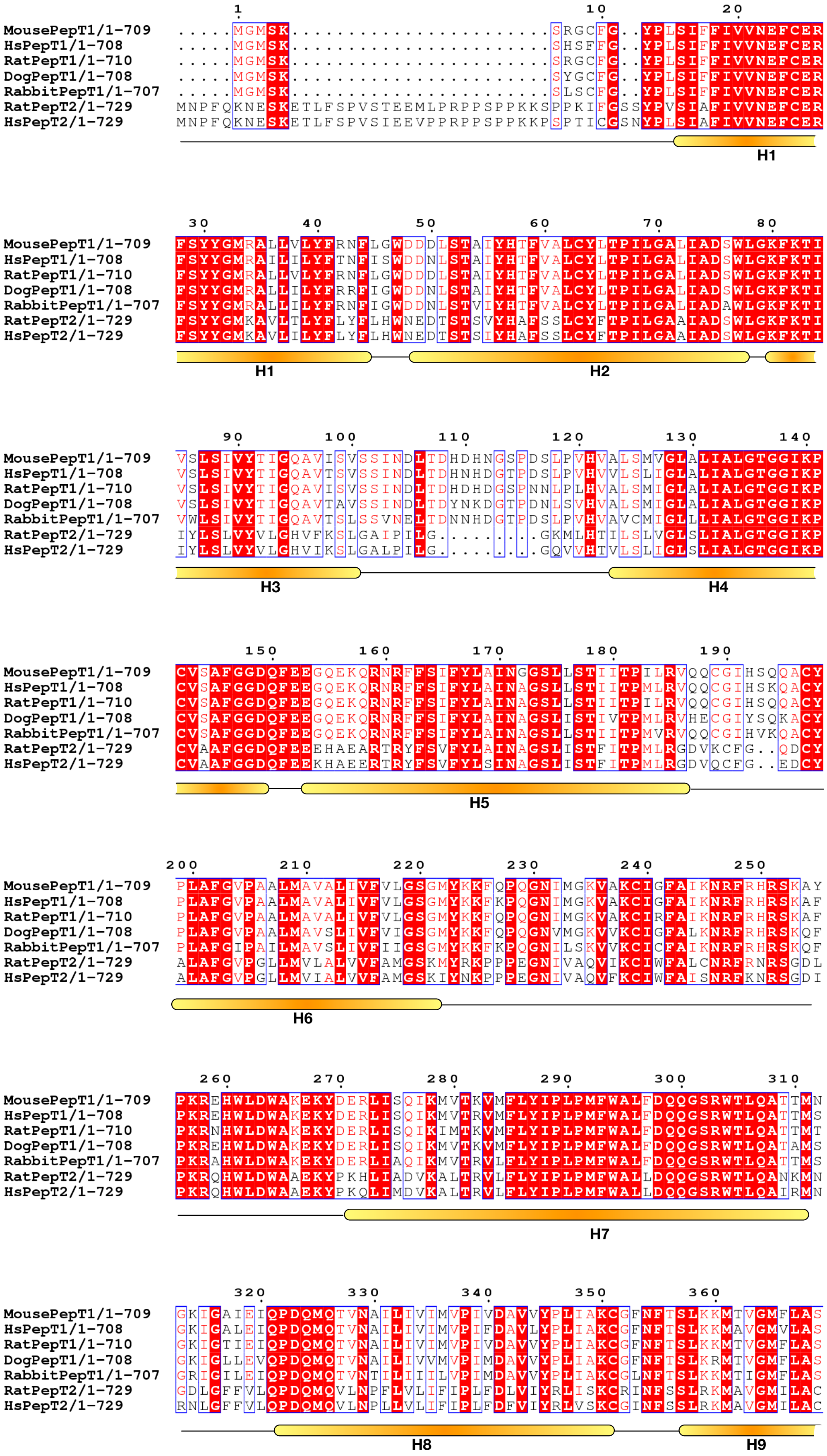
MousePepT1/1-709
HsPepT1/1-708
RatPepT1/1-710
DogPepT1/1-708
RabbitPepT1/1-707
RatPepT2/1-729
HsPepT2/1-729
H1
H2
H3
H4
H5
H6
H7
H8
H9

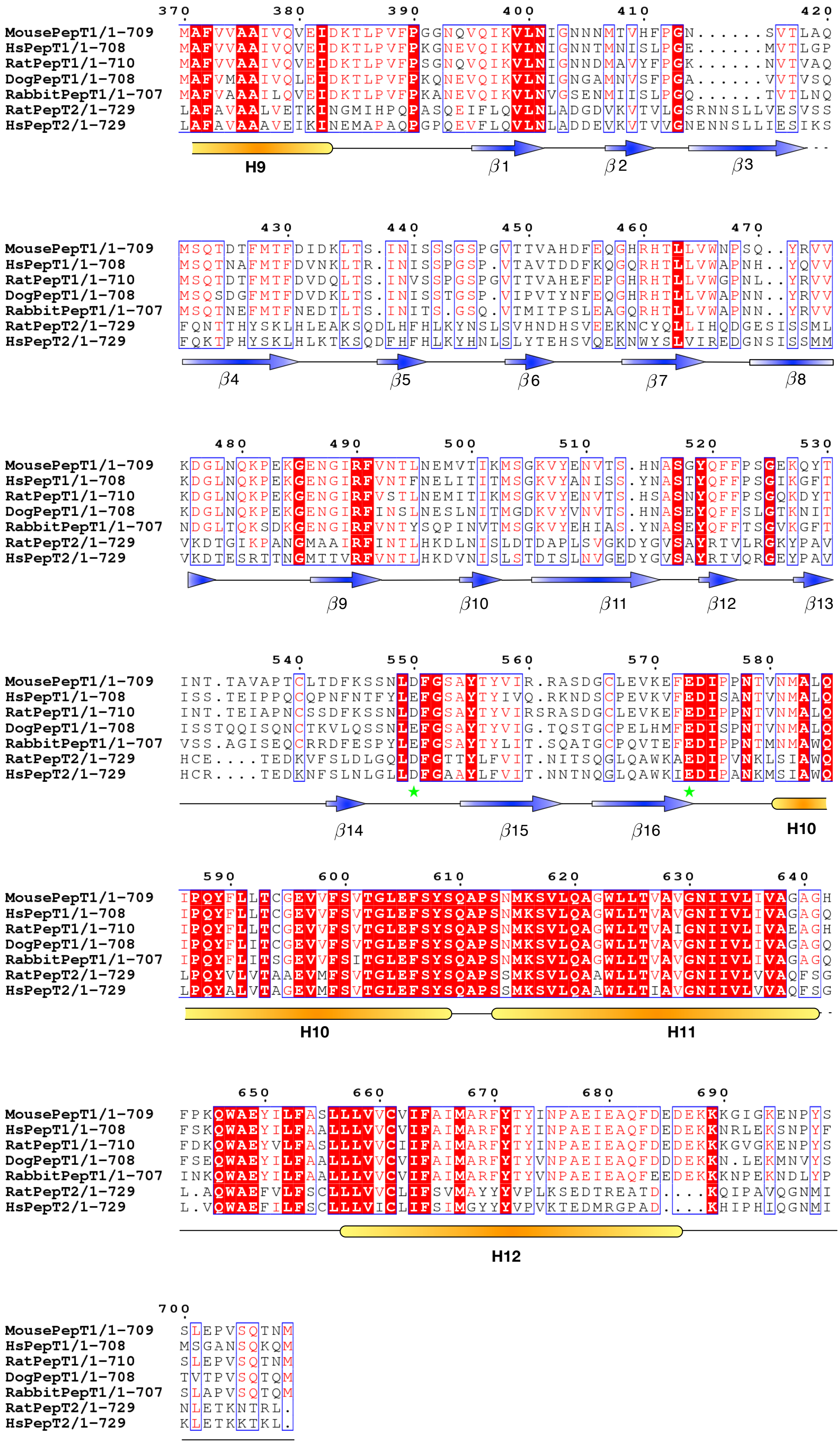
MousePepT1/1-709
HsPepT1/1-708
RatPepT1/1-710
DogPepT1/1-708
RabbitPepT1/1-707
RatPepT2/1-729
HsPepT2/1-729
H9
β1
β2
β3
β4
β5
β6
β7
β8
β9
β10
β11
β12
β13
β14
β15
β16
H10
H11
H12

Figure S1, related to Figure 1 - Sequence alignment and secondary structure of the mammalian POT family peptide transporters.

An amino-acid sequence alignment between the mammalian peptide transporters; mouse PepT1 (Q9J1P7), human PepT1 (P46059), Rat PepT1 (Q75YE4), Dog PepT1 (Q8WMX5), Rabbit PepT1 (P36836), Rat PepT2 (Q63424) and human PepT2 (Q16348). The sequences were aligned using CLUSTALW as implemented in Jalview (Clamp et al., 2004; Waterhouse et al., 2009) and the figure produced using ESPript (Robert and Gouet, 2014). Identical residues are highlighted in red. The locations of the trans-membrane α-helices were determined following sequence alignments with the bacterial POT family transporter PepTSo (Newstead et al., 2011) and are shown as orange tubes below the alignment. The secondary structure of the extra-cellular domain region, as reported here, is highlighted as arrows. Green stars highlight the two residues identified as interacting with trypsin.

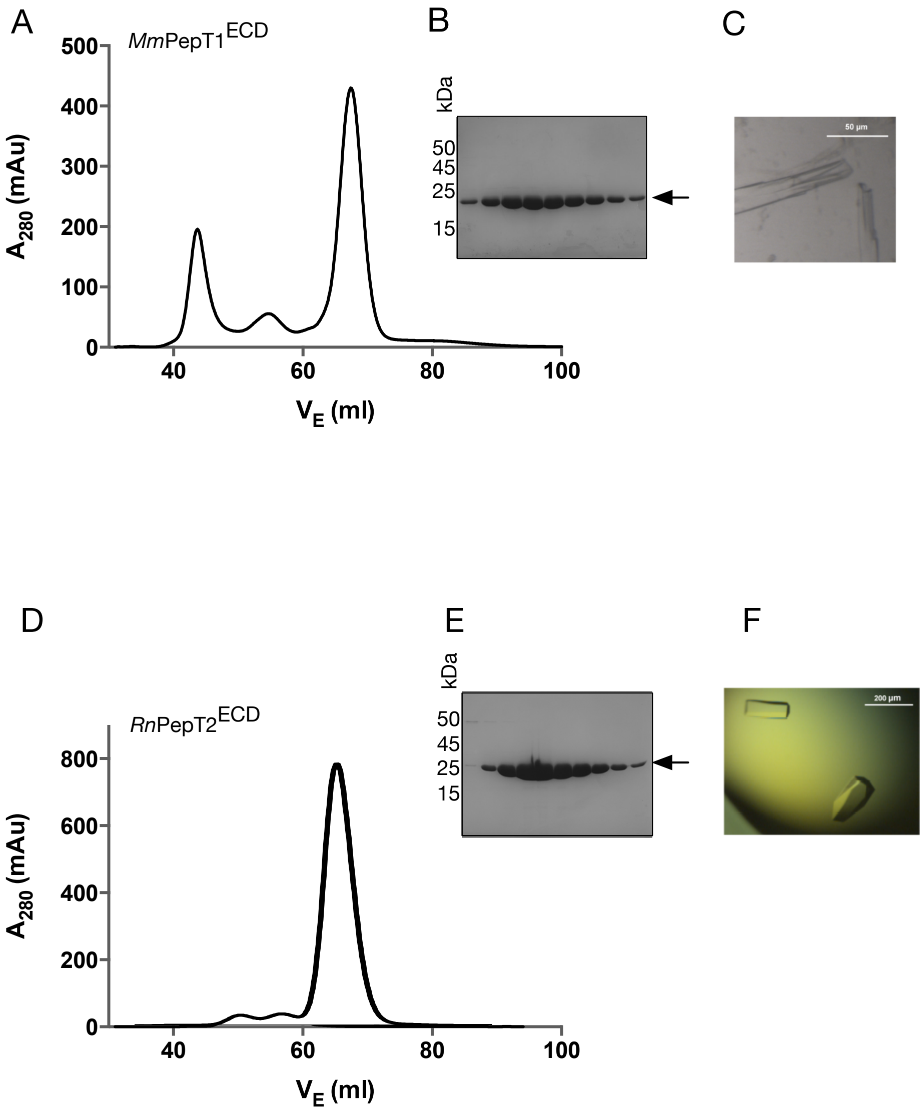

Figure S2, related to Figure 2 - Purification and crystallization of *Mm*PepT1^{ECD} & *Rn*PepT2^{ECD}.

A-C. The final step in the *Mm*PepT1^{ECD} purification showing the UV trace from the S75 16/60 sixe exclusion column. A 15 % Tris-Gly SDS PAGE showing the peak fractions and a representative crystal obstained from the hanging drop vapor diffusion plates. **D-F.** The same data for *Rn*PepT2^{ECD}.

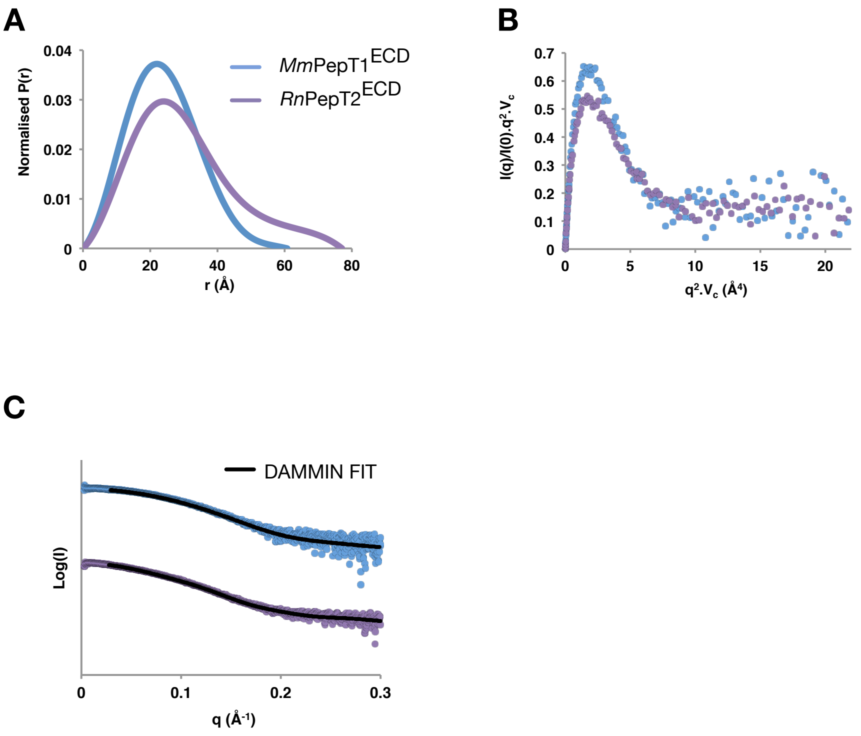

Figure S3, related to Figure 3 – SAXS data analysis of *Mm*PepT1^{ECD} and *Rn*PepT2^{ECD} (shown in blue and purple respectively).

A. Scattering pair distribution functions of the PepT1^{ECD} and PepT2^{ECD} calculated using ScÅtter and normalized to the sum of the paired distances. The distributions shown an increase in the PepT2 D$_{MAX}$ and a shift anyway from the spherical shape observed for PepT1^{ECD} to a wider, more elongated shape. **B.** The dimensionless V_c based Kratky plot (Rambo and Tainer, 2011, 2013) of the ECDs data curves. The plot clearly shows a reduction in the main peek height for *Rn*PepT2^{ECD} compared to *Mm*PepT1^{ECD} indicating an increase the surface area to volume ratio and therefore a large particle. **C.** Shows the stacked scattering curves of the ECDs with the plotted DAMMIN fits of the averaged spherical harmonic models shown in Figure 3C. The DAMMIN χ^2 fits for PepT1^{ECD} and PepT2^{ECD} were 1.09 and 1.27 respectively.

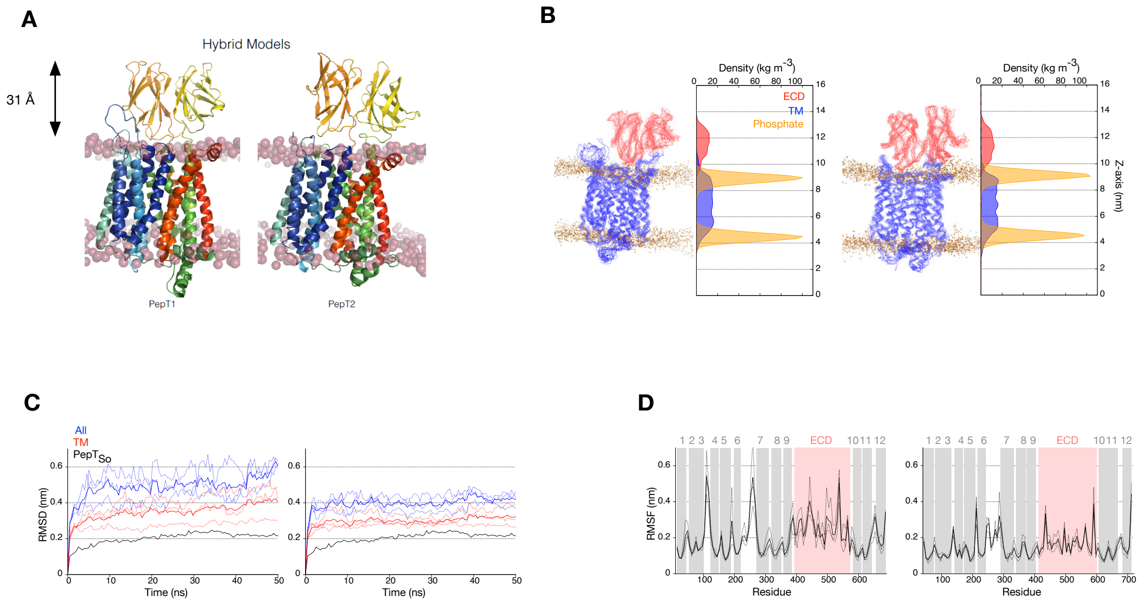

Figure S4, related to Figure 4 - Homology models of human PepT1 and PepT2. **A.** Cartoon representation of the homology models of the human transporters. The trans membrane domains are coloured blue to red, with the extracellular domains shown in yellow. In each subsequent panel PepT1 is on the right and PepT2 the left. **B.** Each hybrid model was inserted into an equilibrated 381-molecule POPC bilayer using GROMACS g_membed protocol. The simulations were run for 50 ns and clearly showed the ECD remaining in the upright position, away from the membrane and the phospholipid head groups**.** **C.** Simulations were analyzed using g_rmsd and g_rmsf tools in GROMACS and showed the models were stable. As a control the crystal structure of PepTSo is shown for reference (black line). **D.** The RMSF analysis shows that the ECD structure is stable and has similar backbone fluctuations to the homology model of the transmembrane domain.

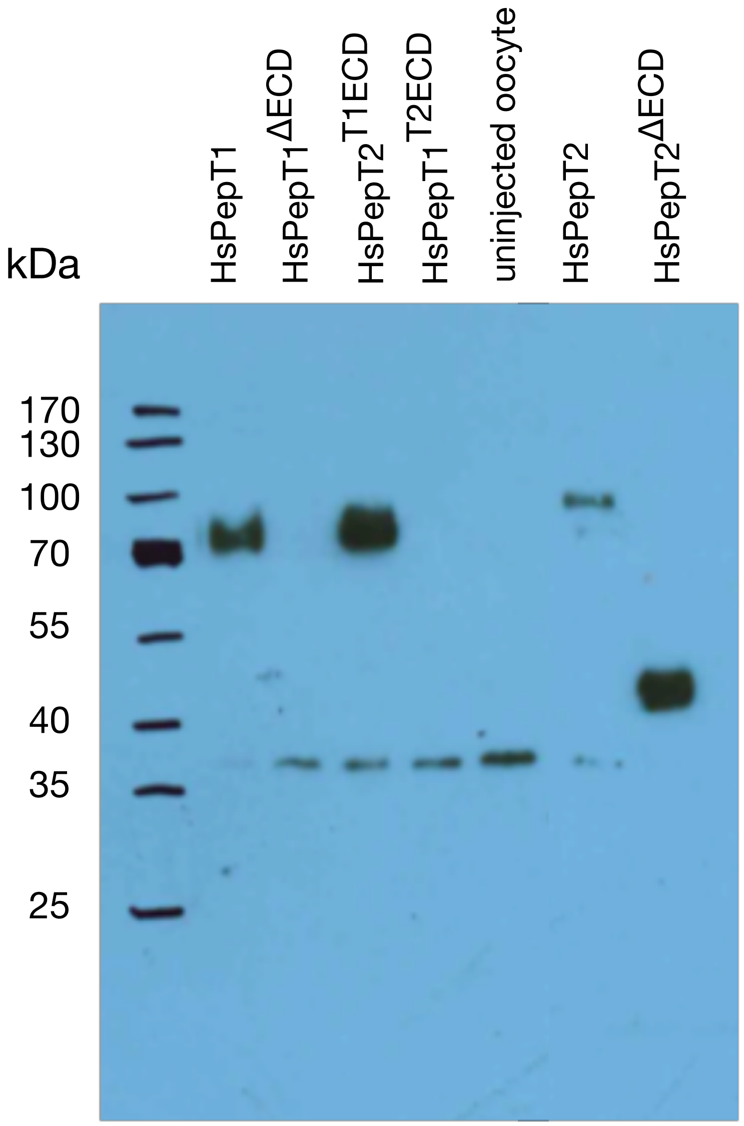

Figure S5, related to Figure 4 - Relative expression levels of *Hs*PepT1 and *Hs*PepT2 constructs in *Xenopus leavis* oocytes. Western blot of a 10% Tris-Gycine SDS-PAGE gel probed using an anti-FLAG antibody. Each lane contains 5 Xenopus eggs after 4 days incubation with either injected mRNA or water control. A cross-reactive band at ~38 kDa was observed in some of the eggs. *Hs*PepT2$^{\Delta ECD}$ runs ~ 10 kDa smaller than the expressed protein, most likely due to known faster migration of transmembrane proteins in SDS-PAGE gels.

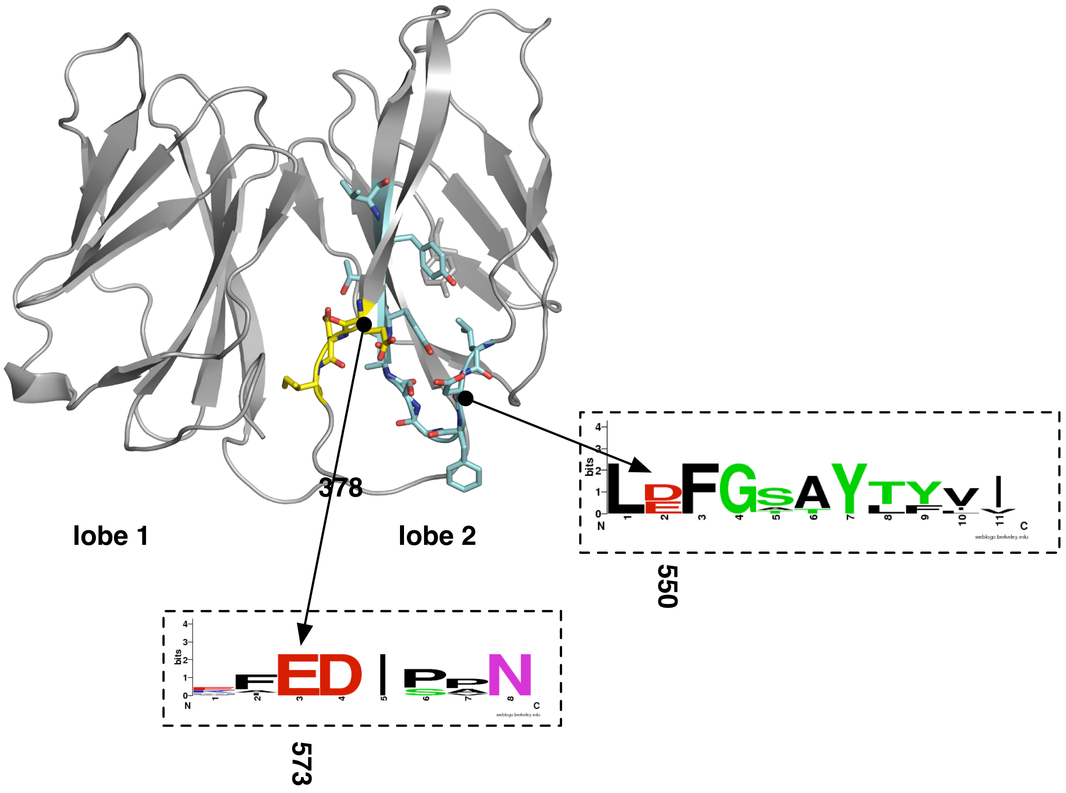

Figure S6, related to Figure 5 – Conserved motifs in the extracellular domains of the mammalian PepT1 and PepT2 proteins. The residues identified as playing an important role in binding to the trypsin protease are located in very conserved regions of the primary structure. Illustrated here the crystal structure of the mouse PepT1^{ECD} as it is thought to exist in solution. Highlighted are the two conserved regions of the sequence, in cyan the region containing the first acidic residue D550 and in yellow the region containing the second acidic residue, E573. Of note is that the second residue in this motif, D574, form part of the salt bridge network identified as playing a role in stabilizing the interface between lobe1 and lobe 2 of the ECD in solution.

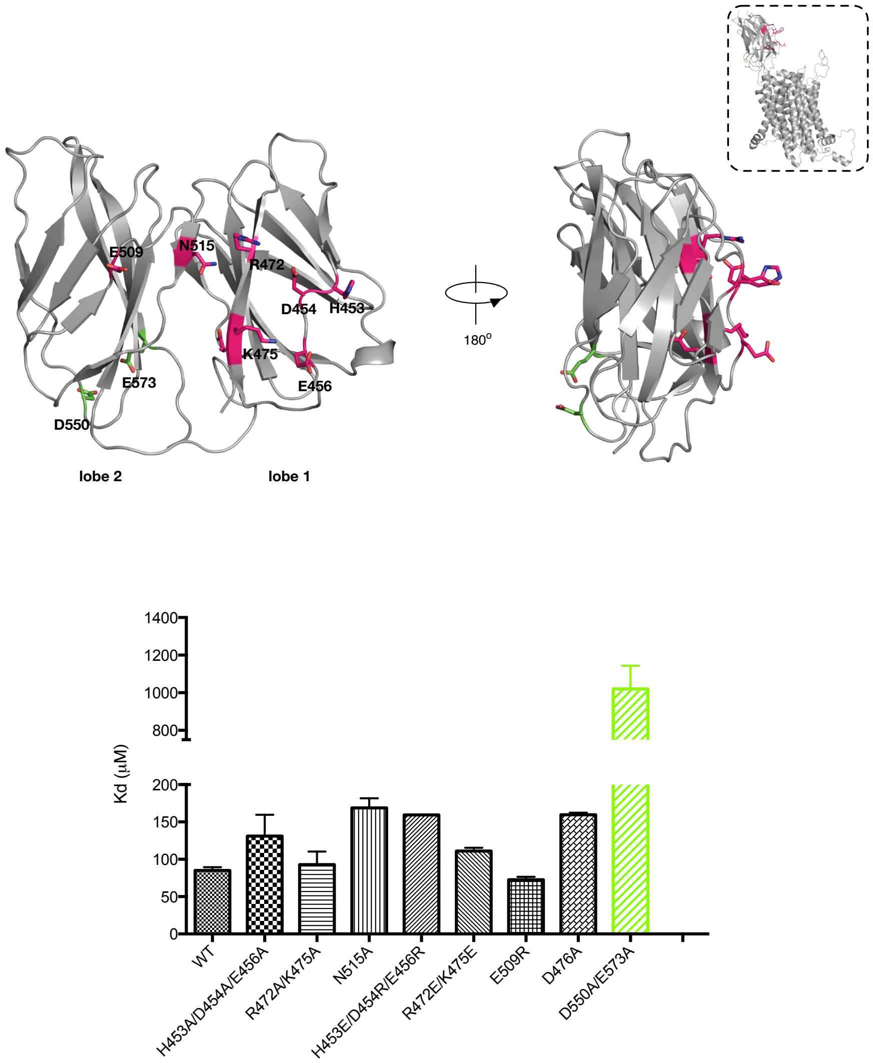

Figure S7, related to Figure 5 – Analysis of binding sites for trypsin on mouse PepT1 ECD. Residues on both the front (magenta) and back (green) face of the mouse PepT1 ECD were analyzed for their interaction with bovine trypsin using SPR. Only residues on the back face of the ECD (D550/E573) resulted in a significant reduction of binding affinity compared to WT. D476A forms part of the salt bridge network stabilizing the interface between lobe1 and lobe 2. Top right - homology model of full length mouse PepT1 showing the location of the residues analyzed with respect to the trans membrane helices and peptide translocation pathway.

Supplementary References:

Clamp, M., Cuff, J., Searle, S.M., and Barton, G.J. (2004). The Jalview Java alignment editor. Bioinformatics (Oxford, England) *20*, 426-427.

Newstead, S., Drew, D., Cameron, A.D., Postis, V.L.G., Xia, X., Fowler, P.W., Ingram, J.C., Carpenter, E.P., Sansom, M.S.P., Mcpherson, M.J., *et al.* (2011). Crystal structure of a prokaryotic homologue of the mammalian oligopeptide-proton symporters, PepT1 and PepT2. The EMBO journal *30*, 417-426.

Rambo, R.P., and Tainer, J.A. (2011). Characterizing flexible and intrinsically unstructured biological macromolecules by SAS using the Porod-Debye law. Biopolymers *95*, 559-571.

Rambo, R.P., and Tainer, J.A. (2013). Super-resolution in solution X-ray scattering and its applications to structural systems biology. Annu Rev Biophys *42*, 415-441.

Robert, X., and Gouet, P. (2014). Deciphering key features in protein structures with the new ENDscript server. Nucleic Acids Res *42*, W320-324.

Waterhouse, A.M., Procter, J.B., Martin, D.M., Clamp, M., and Barton, G.J. (2009). Jalview Version 2--a multiple sequence alignment editor and analysis workbench. Bioinformatics *25*, 1189-1191.
